# Bu-shen-he-mai-fang (HMF) Decoction Inhibits Atherosclerosis by Improving Antioxidant and Anti-Inflammatory Activities in ApoE-deficient Mice

**Published:** 2014-12

**Authors:** Qingqing Hao, Xu Chen, Xiaoming Zhou, Xiaoyu Wang, Xinran Cao, Xingjuan Chen, Yuehua Jiang, Feng Lu, Ke You, Chuanhua Yang, Bo Dong

**Affiliations:** 1 Department of Cardiology, Shandong Provincial Hospital Affiliated to Shandong University, Jinan, Shandong, China;; 2 The Key Laboratory of Cardiovascular Remodeling and Function Research, Shandong University Qilu Hospital, Jinan, Shandong, China;; 3 Department of Pathophysiology, Fenyang College Shanxi Medical University, Fenyang, Shanxi, China;; 4 Department of Cardiology, Affiliated Hospital of Shandong University of Traditional Chinese Medicine, Jinan, Shandong, China

**Keywords:** Chinese medicine, Inflammation, Atherosclerosis

## Abstract

**Objective:**

To observe the effects of Bu–shen- he- mai- fang (HMF) on experimental atherosclerosis in ApoE-deficient mice.

**Materials an Methods:**

Thirty male ApoE-deficient mice were randomly divided into 3 groups (10 mice per group) as follows: one group received the standard high- cholesterol diet (high- cholesterol group, HC); Another group received high- cholesterol diet supplemented with HMF decoction 1.37 g/kg/day; the third group received a high- cholesterol diet, supplemented with atrovastatin 5 mg/kg/day for 8 weeks. The extent of atherosclerosis, the expression of LOX-1 protein and macrophage infiltration were evaluated by H&E, oil red O staining, and immunohistochemical staining. SOD was also measured by a spectrophotometer.

**Results:**

The degree of atherosclerosis was significantly lower in HMF group and atrovastatin group than that in high-cholesterol group. The expression of LOX-1 protein and macrophage filtration were significantly lower in HMF group and atrovastatin group than that in high-cholesterol group. Also, the SOD was higher in HMF group and atrovastatin group than that in high-cholesterol group.

**Conclusion:**

The results suggested that HMF significantly inhibited early atherosclerotic lesions by inhibiting inflammatory response and decreasing the generation of ROS.

## INTRODUCTION

Atherosclerosis is the basis of stroke, coronary heart disease and myocardial infarction. It has been proved that atherosclerosis is based on endothelial cell damage, and is characterized by chronic inflammation of the vascular pathological process. Currently, there are no effective methods for treating atherosclerosis. However, pharmaceutics of traditional Chinese medicine has been undergoing rapid development in China, and now, more traditional Chinese medicine compound and herbal compound extracts are being administered successfully in the treatment of atherosclerosis and coronary heart diseases (1, 2). Clinical practice has proved that Chinese medicinal herbs are efficient in the treatment of chronic diseases. With the development of modern medicine, it has been demonstrated that Chinese medicine not only treats disease in many ways, but also has definite curative effects (3, 4), and that the intervention effect of traditional medicine is multiple targets and with less side effects (3, 4).

Our previous studies found that Bu–shen- he- mai- fang (HMF) can decrease blood cholesterol level, improve the endothelial cell function and inhibit myocardial fibrosis in hypertensive rat (5). Clinical studies have demonstrated that HMF could inhibit left ventricular hypertrophy in patients with hypertension (6, 7). These previous studies indicated that HMF plays a role in treatment of cardiovascular disease. However, the mechanisms involved and whether HMF can inhibit the evolution of atherosclerotic plaque remain unclear. This study was designed to test the hypothesis that HMF can inhibit the evolution of atherosclerotic plaque by its anti-inflammatory and anti-oxygen radical mechanisms.

## EXPERIMENTAL MATERIALS AND METHODS

### Components of Bu-shen-he-mai-fang (HMF)

HMF powder was provided by Jiangsu Tianyin Pharmaceuticals (Jiangsu, China). The herbal drugs were authenticated and standardized according to the Chinese Pharmacopoeia 2005. The HMF is extracted from a group of herbal medicine, which consist of eucommia ulmoides (15 g), astragalus mongholicus (30 g), parasitic loranthus (15 g), rhizoma alismatis (30 g), epimedium brevicornum (30 g), ligustrazine (15 g), Ligustrum lucidum (15 g), earthworm (15 g).

The HMF powder were mixed and decocted twice by distilled water for 1 hour, and the filtrates was obtained and concentrated. The whole processes were manipulated by pharmaceutical preparation section, affiliated to the Hospital of Shandong University of Traditional Chinese Medicine, Jinan, China.

### Foundation of animal model and drug treatment

Male ApoE-deficient mice on a C57BL/6 background (24 weeks old) were obtained from Beijing University Animal Research Center. The mice were kept in separate cages and allowed to acclimatize for 1 week before the study started, and then fed with a high- cholesterol diet (0.25% cholesterol and 15% cocoa butter). The thirty mice were then randomly divided into 3 groups (10 mice per group): one group received the standard high- cholesterol diet (high- cholesterol group, HC), another group received high- cholesterol diet supplemented with HMF decoction 1.37 g/kg/day (the dosage equal to 50 times of 70 kg person) and the third group received a high- cholesterol diet, supplemented with atrovastatin 5 mg/kg bodyweight/ day for 8 weeks. HMF decoction was monitored to ensure that they were completely consumed by the mice.

All animal care and experimental protocols complied with the animal management rule of the Ministry of Public Health, China (document No 55, 2001), and all animal research protocols were approved by the Ethics Committee of Shandong University and the Animal Care Committee of Shandong University.

### Histology and immunohistochemistry

All animal surgeries were carried out under 3% pentobarbital sodium (30 mg/kg, iv) to minimize the pain. The abdominal aorta were removed, fixed overnight in 4% paraformaldehyde, and then dehydrated and embedded in paraffin. Paraffin-embedded arteries were cross sectioned into 4 µm thick pieces, dewaxed, and rehydrated. Serial sections were conventionally stained with H&E. Lipid deposition was identified by oil red O staining. Immunohistochemical techniques were carried out as described previously. Macrophages were identified with the use of a monoclonal antibody (Santa Cruz Biotechnology, Santa Cruz, CA, USA). LOX-1 was identified by use of a purified polyclonal goat anti-mouse antibody (Santa Cruz Biotechnology, Santa Cruz, CA, USA). After incubation with biotinylated secondary antibody followed by avidin-biotin amplification, the slides were incubated with 3, 39-diaminobenzidine (DAB).

Quantification of macrophages and LOX-1 expression was conducted by an automated image analysis system (Image-Pro Plus 5.0, Media Cybernetics, USA), and positive staining area was calculated by mean percentage of the lesion area in at least 8 high-power fields (× 400 magnification).

### TheSerum levels of interleukin-1 ß (IL-1ß) and tumor necrosis factor-α (TNF-α) by ELISA

The levels of IL-1ß and TNF-α were measured by a commercial ELISA kit (SPI-BIO, Bretonneux, France) according to the instructions described previously.

### Biochemical assay

Serum levels of total cholesterol (TC) and triglycerides (TG) were obtained by enzymatic assays with use of an automated biochemical analyzer (Roche Hitachi 917, Japan).

### Measurements of SOD and MDA assays

The SOD activity in atherosclerotic plaque was measured according to the method using a kit (NJBC, Nanjing, China). Tetrazolium salt can be made to form a red formazan dye by superoxide radicals generated by xanthine oxidase and hypoxanthine. The red formazan dye was measured and evaluated at the optical density at 550 nm by a spectrophotometer. The SOD activity was expressed as U/mg protein.

The MDA contents were measured according to thiobarbituric acid method. The procedure was carried out according to the manufacturer’s instruction. (NJBC, Nanjing, China) The samples were determined at a wavelength of 546 nm using a spectrophotometer, and the results were expressed in terms of nmol/mg protein.

### Statistical analysis

Data analysis involved use of SPSS 11.5. Data are expressed as mean ± standard deviation (SD). Independent sample t test was used to compare continuous data for between group differences. A *p* value <0.05 was considered statistically significant.

## RESULTS

### HMF decoction attenate the extent of atherosclerotic lesion

**Figure 1:**
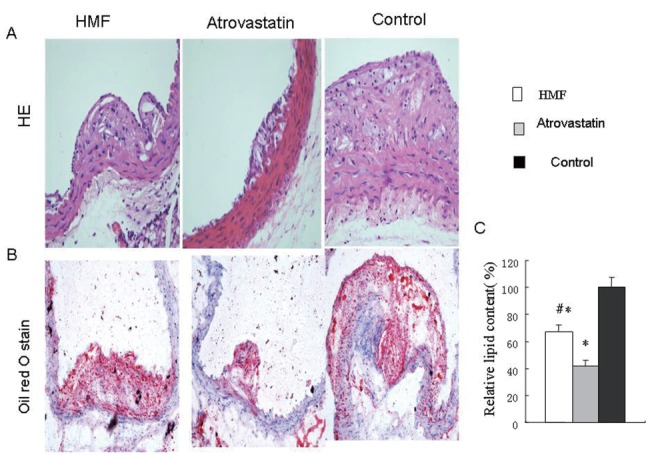
Pathological staining of the atherosclerotic lesions. (A) H&E staining of the atherosclerotic lesions (magnification × 40); (B) Atherosclerotic plaque by oil red O staining (magnification × 40); (C) Quantification of the positive oil red O staining area. ^*^ *p*<0.01, vs. control group. ^#^ *p*<0.01 vs. Atrovastatin group.

To study the effect of HMF and atrovastatin on atherosclerotic evolution, HMF and atrovastatin were administered to ApoE^-/-^ mice. As indicated in Figure 1, HMF and atrovastatin markedly attenuated atherosclerotic lipid content stained by oil red O as compared to high- cholesterol group in ApoE^-/-^ mice (Fig. 1, *p*<0.01). In contrast to atrovastatin group, the lipid content in HMF group was statistically higher than that in atrovastatin group (*p*<0.01).

### Effects of HMF decoction and atrovastatin on macrophage infiltration and LOX-1 expression

**Figure 2:**
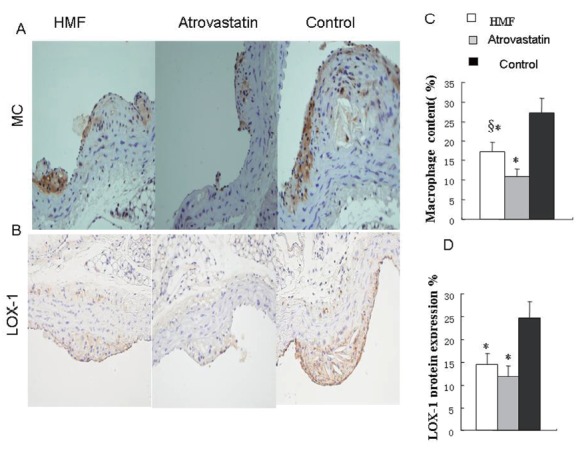
Magrophage and LOX-1 protein expression by immuohistochemistry in atherosclerotic lesions. (A) Magrophage protein expression by immuohistochemisty (magnification × 40); (B) LOX-1 protein expression by immuohistochemisty (magnification × 40); (C) Quantitative analysis of magrophage protein expression. ^*^ *p*<0.01, vs. Control group; ^§^ *p*<0.05 vs. Atrovastatin group; (D) Quantitative analysis of LOX-1 protein expression; ^*^ *p*<0.01, vs. Control group.

The result showed that the macrophage infiltration was statistically lower in HMF group and atrovastatin group than that in high-cholesterol group (Fig. 2A, Fig. 2C, *p*<0.01). In contrast to atrovastatin group, the macrophage infiltration in HMF group was statistically higher than that in atrovastatin group (Fig. 2A, Fig. 2C, *p*<0.01). The LOX-1 protein expression was significantly lower in HMF group and atrovastatin group than that in HC group (Fig. 2B, Fig. 2D, all *p*<0.01), with no significant difference between HMF group and atrovastatin group.

### Effects of HMF decoction and atrovastatin on IL-1ß and TNF-α expression

**Figure 3:**
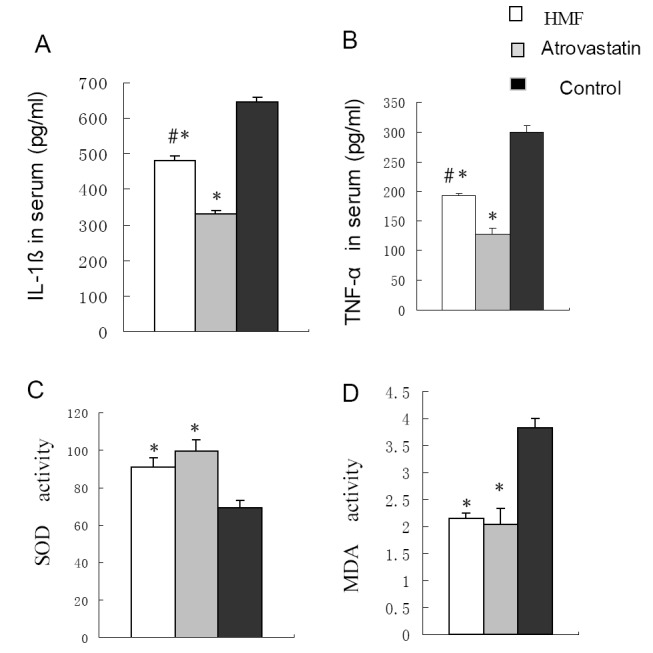
The serum levels of IL-1ß and TNF-α protein and ROS level in ApoE-deficient mice. (A) Level of IL-1ß protein in ApoE-deficient mice. ^*^ *p*<0.01, vs. Control group. ^#^ *p*<0.01 vs. Atrovastatin group; (B) TNF-α level in ApoE-deficient mice. ^*^ *p*<0.01, vs. Control group. ^#^ *p*<0.01 vs. Atrovastatin group; (C) SOD level in ApoE-deficient mice. ^*^ *p*<0.01, vs. Control group. (D) MDA level in ApoE-deficient mice. ^*^ *p*<0.01, vs. Control group.

The serum levels of IL-1ß and TNF-α protein were evaluated by ELISA. The result showed that the levels of IL-1ß and TNF-α were statistically decreased in HMF group and atrovastatin group than that in HC group (Fig. 3A, Fig. 3B, *p*<0.01). Furthermore, the result showed that the levels of IL-1ß and TNF-α were also significantly higher in HMF group than that in atrovastatin group (Fig. 3A, Fig. 3B, *p*<0.01).

### Effect of HMF decoction and atrovastatin on serum lipid levels

At the end of experiment, administration of HMF lowered serum levels of total cholesterol (TC) and Triglyceride (TG) compared with the HC group (Table 1, *p*<0.05). Similarly, administration of atrovastatin significantly lowered serum levels of total TC and TG compared with the HC group (Table 1, *p*<0.01). Furthermore, the levels of total TC and TG were further decreased in atrovastatin group than that in HMF group (Table 1, *p*<0.01).

**Table 1:** Effect of HMF and atrovastatin on Serum Total Cholesterol (TC), Triglyceride (TG) Levels in 3 Groups of Mice

Groups	TC (mg/dL)	TG (mg/dL)

HMF	571.91 ± 59.26^b^^c^	245.46 ± 24.92^b^^c^
Atrovastatin	353.16 ± 53.21^a^	191.59 ± 17. 67^a^
Control	687.88 ± 95.60	320.79 ± 20.77

a *P*<0.01 *vs.* Control group;

b *P*<0.05 *vs.* Control group;

c *P*<0.01 *vs.* Atrostation group.

### ROS level in three groups

To evaluate the antioxidant effect of HMF in our study, we measured the level of SOD and MDA. The result showed that the serum levels of SOD in HC group decreased. In contrast, the level of SOD all increased in HMF group and atrovastatin group as compared with that of HC group (Table 2, Fig. 3C, *P*<0.01). In addition, the level of MDA was increased in HC group as compared with those in HMF group and atrovastatin group (Table 2, Fig. 3D, *P*<0.01). There is no statistically significant difference in the levels of SOD and MDA between HMF group and atrovastatin group.

**Table 2:** The levels of SOD and MDA in 3 Groups of Mice

Groups	SOD (U/mg protein)	MDA (nmol/mg protein)

HMF	90.94 ± 5.26^a^	2.15 ± 1.01^a^
Atrovastatin	99.42 ± 6.31^a^	2.05 ± 0.29^a^
Control	69.54 ± 3.72	3.84 ± 0.16

a *P*<0.01 vs. Control group.

## DISCUSSION

The main finding of this study was that HMF markedly attenuated atherosclerotic evolution, inhibited the macrophage infiltration and LOX-1 expression, and decreased the levels of IL-1ß and TNF-α expression in apoE-deficient mice. In addition, the HMF markedly increased the levels of SOD and decreased the levels of TC and TG, and the effects of these were similar to atrovastatin treatment. To the best of our knowledge, our study is the first to show that HMF inhibited atherosclerosis and have the similar protection effect to atrovastatin in terms of lipid-lowering, anti-inflammatory, and anti-oxidatory effects in apoE-deficient mice.

HMF, derived from a group of herbal medicine including eucommia ulmoides, astragalus mongholicus, parasitic loranthus, rhizoma alismatis, earthworm, epimedium brevicornum, ligustrazine and ligustrum lucidum, has been used to treat patients with hypertension, coronary heart disease in clinical practice. Previous studies have indicated that HMF has pleiotropic effects including improvement of endothelial function, lipid lowering, and inhibition of myocardial fibrosis. Eucommia ulmoides is the main ingredient of HMF. It is one of the most popular tonic herbs in Asia, especially in China. It is not only used as a traditional herb medicine, but also used as a food therapy. Recent study showed that seven main antioxidants from eucommia ulmoides oliv had been demonstrated, indicating that eucommia ulmoides oliv has an important antioxidant activity (7). It is found that eucommia ulmoides extracts have many pharmacological actions, especially in treating hyperlipidemia, diabetes, obesity, hypertension, aging and in increasing longevity. Eucommia ulmoides has important protective effects in various lipid peroxidation models. It can reduce oxidative damage of biomolecule and has the ability to regulate ER stress (8). Study showed that eucommia ulmoides extracts could protect liver against CCl4-induced hepatic lipid accumulation (9). Studies have shown that eucommia ulmoides leaf extracts has potent protective effects in various lipid peroxidation models and reduce oxidative damage of biomolecules. Astragalus mongholicus has long been used to treat heart disease in Chinese traditional medicine. Modern study showed that it has anti-oxidant and anti-inflammatory ability. It can protect the endothelial cell function and inhibit atherosclerosis formation (10). Similarly, It has been reported that parasitic loranthus has antioxidative,and hyperglycemic-lowering effects and can reduce body weight (11). Ligustrum robustum extracts also showed remarkable antioxidant capacity to scavenge free radicals and anti-inflammatory activity *in vitro* (12). Several studies found that earthworm has antioxidant, anticoagulant,and lipid lowering ability. Study showed that rhizoma alismatis could reduce inflammation by suppressing NF-κB activation, decrease blood lipid level, and prevent cardiovascular complications. Dan et al found that administration of alismatis rhizome treatment give rise to significant decrease in cholesterol, triglyceride and serum high-density lipoprotein cholesterol in hyperlipidemic mice. Ligustrazine has many effects on cardiovascular diseases, including endothelial protective function, anti-myocardial ischemia and anti-ischemic reperfusion injury effects. Collectively, all these pharmacological effects could contribute to protect endothelial cell activity and inhibit atherosclerosis and treat coronary heart disease by its anti antioxidant , anti inflammatory abilities and decreasing blood lipid level and so on.

Recent study showed that lectin-like oxidized low-density lipoprotein receptor 1 (LOX-1) plays a key role in the pathogenesis of atherosclerotic plaque initiation, formation and rupture (13, 14). LOX-1 is the mark of early endothelial cell dysfunction, and it takes part in endothelial dysfunction, monocyte adhesion, inflammatory response, foam cell formation as well as plaque instability. Some study have showed that LOX-1 increases the instability of atherosclerotic plaques, takes part in the ultimate clinical sequelae of plaque rupture and is involved in the pathogenesis of life-threatening myocardium ischemia (15, 16). It has been showed that LOX-1 protein expression is intensively expressed in vulnerable plaques with macrophage-rich lipid core areas and thin fibrous caps. Also, overexpression of LOX-1 in ApoE-deficient mice accelerates the pathogenesis of atherosclerotic plaque and produces inflammatory response (17). Further study showed that LOX-1 modulates matrix metalloproteinases (MMP) activity, apoptosis of smooth muscle cells (SMC), and collagen content, all of which take part in the pathogenesis of vulnerable plaque and the occurrence of acute coronary syndrome (17, 18). Inhibition of LOX-1 expression by drugs including angiotensin-converting enzyme inhibitors (ACEI), angiotensin receptor blocker (ARB) and statin have been demonstrated to protect endothelial cell function, prevent evolution of atherosclerotic plaque and increase stability of plaque. Our previous report showed that the AT1 receptor antagonist losartan significantly inhibited the expression of LOX-1 and the progression of atherosclerotic plaque (19). Our study also showed that inhibition of ACE activity by overexpression of ACE2 can decrease LOX-1 expression, prevent atherosclerotic evolution and increase the stability of atherosclerotic plaque (20). In this study, we found that not only atrovastatin, but also HMF significantly attenuated LOX-1 expression and macrophage infiltration, indicating that HMF plays an important role in the protection of endothelial cell activity and inhibition of atherosclerosis, and thus exhibiting the same effects as atrovastatin. In addition, we speculated that HMF may have a protective role through decreasing the vulnerability of atherosclerotic plaque for which further research is necessary.

It has been previously demonstrated that LOX-1 expression can be modulated by many stimuli related to atherosclerosis, including proinflammatory cytokines such as interleukin-1 (IL-1) and tumor necrosis factor alpha (TNF-α) , angiotensin II and oxidized low density lipoprotein (OX-LDL) (16) . So we speculated that mechanism of HMF against LOX-1 expression may be due to its lipid lowering and anti-inflammatory effects.

Inflammation occurs in all stages of atherosclerosis (21). Macrophage is the main inflammatory cell in atherosclerotic plaques. It can produce matrix metalloproteinases (MMPs), which degrade the extracellular matrix, lead to plaque disruption and acute arterial thrombosis and give rise to the occurrence of ACS (22). Activated inflammatory cells within plaques release cytokines, such as MCP-1, IL-1 and TNF-α, which further produce plaque inflammatory response and promote matrix degradation and plaque rupture. A lot of studies have demonstrated that MCP-1, IL-1 and TNF-α levels increased in atherosclerosis. IL-1 and TNF-α could induce oxidation of LDL and promote aggregation of oxidized LDL, and then the oxidized LDL can induce local vascular cells to produce MCP-1, which can promote monocyte recruitment and increase the inflammatory response and enhance the progression of atherosclerosis. Therefore, IL-1 and TNF-α are important inflammatory cytokines in the pathogenesis of atherosclerotic evolution. Our result showed that the levels of IL-1β and TNF-α were significantly higher in atherosclerotic plaque. Conversely, HMF and atrovastatin decreased the level of IL-1β and TNF-α and this result indicated that HMF played an important anti-inflammatory role in the treatment of AS.

It is reported that reactive oxygen species (ROS) influences cell growth and apoptosis; induces inflammatory response and is involved in the pathogenesis of atherosclerosis (23). ROS also promotes the expression of adhesion molecules and chemokines, e.g. MCP-1. All these pathogenesis contribute to atherosclerosis, coronary heart disease, diabetes and other chronic diseases. There is a growing interest in natural antioxidants found in plants. Many studies found that herbs medicine have an antioxidant effect. Studies have demonstrated that herbs, for example, eucommia ulmoides, parasitic loranthus, ligustrum robustum and astragalus mongholicus all have antioxidant activity (7, 11). To test whether the HMF improves the levels of antioxidant enzymes, we measured the levels of SOD and MDA, and the results showed that the SOD levels were significantly increased in the HMF than that in the HC group, suggesting that HMF boosts the activity of antioxidant enzymes. This may be one of important mechanisms in treatment of atherosclerosis.

In summary, our study demonstrated that HMF significantly inhibited early atherosclerotic lesions by decreasing ROS generation and inhibiting inflammatory response and decreasing lipid level.
